# Downsizing a giant: re-evaluating *Dreadnoughtus* body mass

**DOI:** 10.1098/rsbl.2015.0215

**Published:** 2015-06

**Authors:** Karl T. Bates, Peter L. Falkingham, Sophie Macaulay, Charlotte Brassey, Susannah C. R. Maidment

**Affiliations:** 1 Department of Musculoskeletal Biology, University of Liverpool, Duncan Building, Daulby Street, Liverpool L69 3GE, UK; 2 School of Natural Sciences and Psychology, Liverpool John Moores University, James Parsons Building, Bryon Street, Liverpool L3 3AF, UK; 3 Faculty of Life Sciences, University of Manchester, Manchester M13 9PL, UK; 4 Department of Earth Science and Engineering, Imperial College, South Kensington, London SW7 2AZ, UK

**Keywords:** *Dreadnoughtus*, body mass, modelling, scaling equations

## Abstract

Estimates of body mass often represent the founding assumption on which biomechanical and macroevolutionary hypotheses are based. Recently, a scaling equation was applied to a newly discovered titanosaurian sauropod dinosaur (*Dreadnoughtus*), yielding a 59 300 kg body mass estimate for this animal. Herein, we use a modelling approach to examine the plausibility of this mass estimate for *Dreadnoughtus*. We find that 59 300 kg for *Dreadnoughtus* is highly implausible and demonstrate that masses above 40 000 kg require high body densities and expansions of soft tissue volume outside the skeleton several times greater than found in living quadrupedal mammals. Similar results from a small sample of other archosaurs suggests that lower-end mass estimates derived from scaling equations are most plausible for *Dreadnoughtus*, based on existing volumetric and density data from extant animals. Although volumetric models appear to more tightly constrain dinosaur body mass, there remains a clear need to further support these models with more exhaustive data from living animals. The relative and absolute discrepancies in mass predictions between volumetric models and scaling equations also indicate a need to systematically compare predictions across a wide size and taxonomic range to better inform studies of dinosaur body size.

## 1. Introduction

Sauropod dinosaurs include the largest terrestrial animals to have ever evolved, and mass properties are regarded as a crucial component of their functional, behavioural and evolutionary dynamics [1]. Recently, Lacovara *et al.* [2] described a gigantic, near-complete titanosaurian sauropod, *Dreadnoughtus schrani*, from Argentina. These authors used a scaling relationship between long bone (femoral plus humeral) circumference and body mass [3] to derive a mass estimate of 59 300 kg for the holotype of *Dreadnoughtus*. This scaling equation is well supported statistically in living tetrapods and to date has been used to estimate the body mass of extinct taxa to facilitate studies of physiology and growth (e.g. [4]) and macroevolutionary dynamics [1]. However, the mass estimate seems high given that in overall skeletal proportions *Dreadnoughtus* only marginally exceeds those of near-complete specimens of other sauropods (e.g. *Apatosaurus* and *Giraffatitan*) whose masses have been estimated at 25–35 000 kg by various methods (e.g. [3,5]). In this paper, we use a digital three-dimensional skeletal model and volumetric reconstructions to directly examine the plausibility of the 59 300 kg mass estimate for *Dreadnoughtus*, and subsequently comment upon the use of scaling equations to estimate dinosaur body mass.

## 2. Material and methods

**Figure 1.**
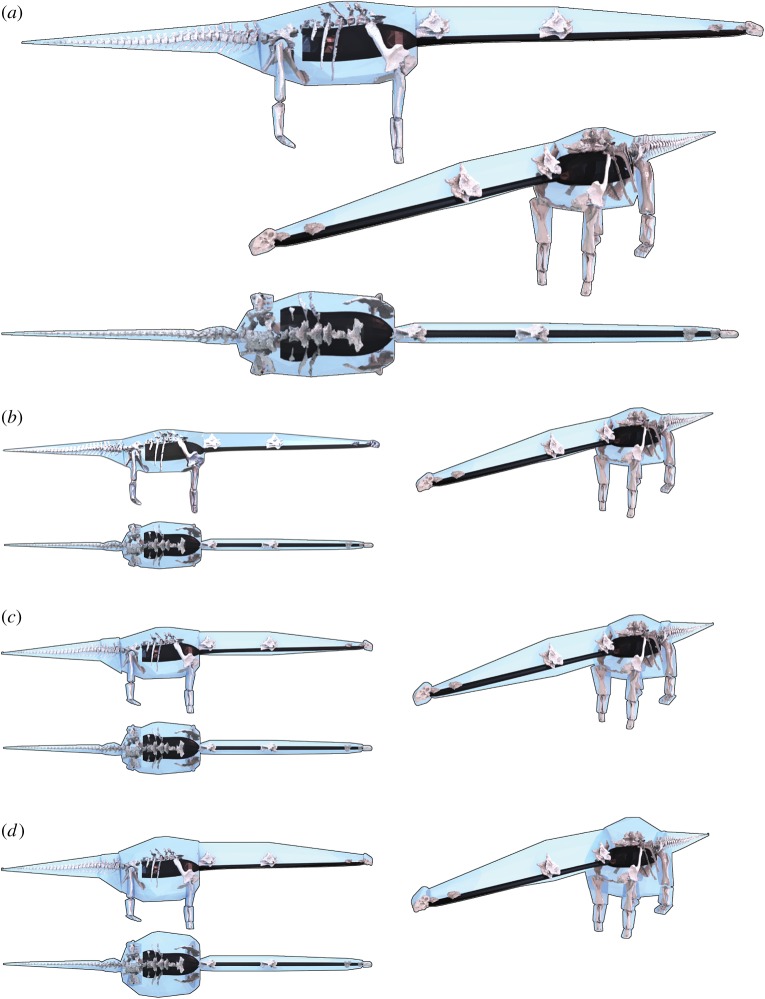
*Dreadnoughtus* three-dimensional skeletal model and the (*a*) convex hull, (*b*) plus 21%, (*c*) maximal and (*d*) scaling equation mass volumetric reconstructions in lateral, oblique and aerial views. Black structures are respiratory volumes. (Online version in colour.)

A digital model of the *Dreadnoughtus* skeleton from Lacovara *et al.* [2] was used as a basis for a three-dimensional volumetric model (figure 1). For comparative purposes, we also modelled six extant taxa (three birds, two crocodilians and one lizard) and two other large sauropods using identical methods: *Giraffatitan brancai*, based on a laser scan of MB (Museum für Naturkunde, Berlin, Germany) SII from our previous study [5], and *Apatosaurus louisae*, based on a new three-dimensional model of CM (Carnegie Museum, USA) 3018 generated using photogrammetry [6]. Each three-dimensional skeletal model was posed in a standard ‘neutral’ posture, with the tail and neck extending horizontally and the limbs in a fully extended, vertical position (figure 1). Models were then divided into the following body segments: head, neck, ‘trunk’ (thorax and limb girdles), tail, thigh, shank, foot, humerus, forearm and hand.

The holotype of *Dreadnoughtus* is missing most of the cervical vertebrae, as well the manus, skull and distal tip of the tail. Our convex hulling approach [5] to volumetric reconstruction involves tight-fitting three-dimensional convex polygons to each body segment. As the extent of an object's convex hull is dictated solely by its geometric extremes, we were able to minimize the amount of skeletal reconstruction in our model (electronic supplementary material, figure S1). For the hand and skull, we used photogrammetric models of these elements from *Rapetosaurus* (FMNH PR 2209), another titanosaur, and re-scaled them using the reconstruction in Lacovara *et al.* (fig. 2 in [2]). To allow convex hulling to connect the ‘trunk’ and neck segments, we duplicated the ninth cervical vertebra preserved in the specimen and placed its posterior surface above the most anterior point of pectoral girdle at a height consistent with the position of the preserved dorsal vertebrae. An additional 10% was added to the distal tail using the reconstruction of Lacovara *et al.* [2] as a guide (electronic supplementary material, figure S1). In the electronic supplementary material, we provide extensive sensitivity tests of our skeletal reconstruction procedure (electronic supplementary material, figures S1–S8).

The minimum convex hull volume for each skeletal body segment was calculated using the MATLAB (www.mathworks.com) qhull command [5,8]. The total minimum convex hull volume provides the minimum volume estimate for each animal, and a baseline for our sensitivity analysis in which we generated three further models. In the first model, the minimal convex hulls were geometrically expanded by 21%, following a previous study in which live body mass was estimated to have been on average 21% greater than that calculated from minimum convex hulls for a range of extant mammals [5]. We subsequently generated a ‘maximal mass model’ in which the volume of the trunk segment was increased by 50% and those of all other segments by 100%. Finally, we expanded the minimum convex hull model of *Dreadnoughtus* by the amount required to match the total body masses predicted by the scaling equation of [3]. For the sauropod models, body segments were given an initial density of 1000 kg m^−3^. Zero-density respiratory structures in the head, neck and ‘trunk’ segments were reconstructed and the volumes of these structures subtracted from their overall segment volume, as in previous volumetric studies of dinosaurs [7,9,10]. Homogeneous body densities were used for the extant taxa, based on published values for crocodiles and chickens [10].

## 3. Results

**Figure 2.**
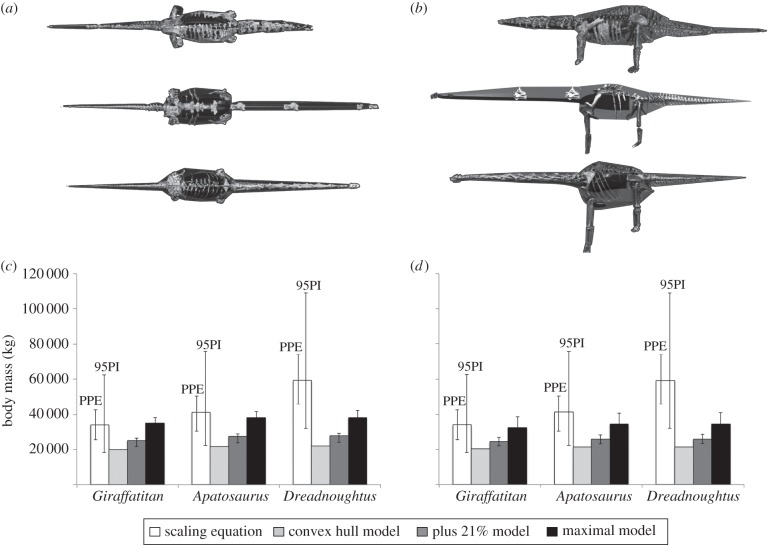
Comparison of skeletal proportions and convex hull volumes for *Apatosaurus* (top), *Dreadnoughtus* (middle) and *Giraffatitan* (bottom) in (*a*) dorsal and (*b*) lateral views. Comparison of mass predictions from the models in this study to masses derived from the scaling equation [2], with (*c*) model mass and density calculated using reconstructed zero-density respiratory structures, and (*d*) density artificially set to 800 kg m^−3^ [7]. The positive error bar on our maximal models represents the mass predicted by expanding convex hull volumes by the highest exponent (×1.91) for mammals [5] and archosaurs to date. The ‘PPE’ error bars on scaling equation outputs represent the average ‘per cent prediction error’, whereas ‘95PI’ error bars represent the ‘95% prediction interval’.

The convex hull volume reconstruction of *Dreadnoughtus* results in a total body volume of 26.910 m^3^ (figure 1*a* and table 1). Expanding this minimum convex hull volume by 21% raises the whole-body volume to 32.534 m^3^ (figure 1*b*), while the volume of our maximal model is 43.016 m^3^ (figure 1*c*). Deducting the volume of our reconstructed respiratory structures from each of these models yields total body masses of 22 117, 27 741 and 38 225 kg for the three model iterations. These data and data from equivalent models of *Apatosaurus* and *Giraffatitan* (figure 2*a*,*b*) are shown in table 1, while the data from extant taxa are tabulated in the electronic supplementary material (tables S1–S6, and figures S8 and S9). Convex hull volumes are available in the electronic supplementary material.

**Table 1.** Mass property data for convex hull reconstructions of *Dreadnoughtus*, *Apatosaurus* and *Giraffatitan*, and summary of whole-body mass data from different model iterations.

	*Dreadnoughtus*			*Apatosaurus*			*Giraffatitan*		
convex hull	volume (m^3^)	density (kg m^−3^)	mass (kg)	volume (m^3^)	density (kg m^−3^)	mass (kg)	volume (m^3^)	density (kg m^−3^)	mass (kg)
body segments									
head	0.033	1000	33.49	0.02	1000	23.46	0.06	1000	59.45
neck	3.110	1000	3109.99	2.62	1000	2615.16	2.46	1000	2461.00
trunk	20.382	1000	20 381.96	20.12	1000	20 187.65	19.85	1000	19 850.92
tail	1.011	1000	1011.35	1.86	1000	1861.20	0.78	1000	774.76
humerus	0.186	1000	186.08	0.23	1000	232.34	0.30	1000	298.78
forearm	0.097	1000	97.36	0.10	1000	103.01	0.16	1000	160.67
hand	0.024	1000	24.11	0.03	1000	25.96	0.09	1000	85.98
humerus	0.186	1000	186.08	0.28	1000	275.31	0.30	1000	298.78
forearm	0.097	1000	97.36	0.10	1000	103.01	0.16	1000	160.67
hand	0.024	1000	24.11	0.03	1000	25.96	0.09	1000	85.98
thigh	0.246	1000	246.13	0.35	1000	351.27	0.29	1000	294.19
shank	0.110	1000	109.86	0.21	1000	208.57	0.19	1000	193.06
foot	0.042	1000	41.91	0.08	1000	84.62	0.04	1000	35.69
thigh	0.246	1000	246.13	0.35	1000	351.27	0.29	1000	294.19
shank	0.110	1000	109.86	0.21	1000	208.57	0.19	1000	193.06
foot	0.042	1000	41.91	0.08	1000	84.62	0.04	1000	35.69
axial total	25.50	1000	24 536.80	24.62	1000	24 687.47	23.15	1000	23 146.13
hind limb total	0.796	1000	795.80	1.289	1000	1288.92	1.046	1000	1045.88
fore limb total	0.614	1000	615.09	0.722	1000	722.62	1.092	1000	1090.87
whole body	26.91	1000	25 947.68	26.63	1000	26 699.01	25.28	1000	25 282.88
respiratory structures									
head	0.003	1000	3.43	0.001	1000	0.99	0.0036	1000	3.60
neck	4.30	1000	4303.67	4.60	1000	4602.86	5.00	1000	5000.39
trunk	0.49	1000	486.48	0.29	1000	291.95	0.33	1000	332.54
model iteration									
minimum convex hull	26.91	821.9	22 117.98	26.63	818.8	21 803.21	25.284	788.8	19 946.35
plus 21% model	32.53	852.7	27 741.68	32.26	850.5	27 363.56	30.54	825.2	25 204.65
maximal model	43.02	888.6	38 224.57	43.08	886.4	38 187.23	40.40	867.9	35 060.42

## 4. Discussion and conclusion

The mass of *Dreadnoughtus* was estimated at 59 300 kg using the raw bivariate predictive equation of Campione & Evans [3]. The masses of our three volumetric reconstructions of *Dreadnoughtus* (figure 1*a*–*c* and table 1) are equivalent to 37, 47 and 64% of the 59 300 kg scaling equation mass. The ‘average per cent prediction error’ from the bi-variate equation gives a minimum mass of 44 095 kg (5780 kg or 15% higher than our ‘maximal’ model) and a maximum mass of 74 487 kg (36 262 kg or 95% higher than our ‘maximal’ model). The ‘95% prediction interval’ from the equation yields a range of 32 000–109 000 kg for *Dreadnoughtus*, which overlaps with model estimates (figure 2).

Convex hulling provides a close, objective approximation of the body volume defined by a skeleton alone [5,8]. A volume 2.38 times larger than that of our convex hull model is required for *Dreadnoughtus* to achieve the mean or ‘best-estimate’ scaling equation mass of 59 300 kg, using our estimates for the size of respiratory structures (figure 1*d*). This represents an expansion more than 6.5 times greater than the average value found in a sample of quadrupedal mammals spanning major taxonomic groups [5]. This 2.38 times expanded model (figure 1*d*) has a bulk density of 925 kg m^−3^, which is higher than any presently published estimate for sauropods (range 791–900 kg m^3^; electronic supplementary material, table S7). If lower-end estimates of 800 kg m^−3^ for sauropod density [7] are correct, then achieving a body mass of 59 300 kg for *Dreadnoughtus* would require body and respiratory volumes of 74.125 m^3^ and 14.825 m^3^, respectively, the latter representing a 310% expansion of our respiratory volumes (figure 1). Filling the entire ribcage with a zero-density respiratory structure (electronic supplementary material, figure S7), which is obviously highly implausible, only produces a 212% increase in respiratory volume. It is clear from our model that bulk densities as low or approaching 800 kg m^3^ cannot be reconciled with a total body mass of 59 300 kg given the skeletal proportions of *Dreadnoughtus* and the space available within the ribcage for low-density respiratory structures.

Comparison of mass predictions from volumetric reconstructions of near-complete skeletons of *Apatosaurus* and *Giraffatitan* (figure 2) to the mean scaling equation masses, produces a qualitatively similar result: scaling equation mass predictions exceed those of our maximal models (figure 2*c*,*d*). The disparity between the two approaches increases further if the whole-body densities of these models are set to lower-end estimates for sauropods (800 kg m^−3^ [7]) rather than predicting density by inclusion of respiratory structures. In the case of both *Apatosaurus* and *Giraffatitan*, there is clear overlap between the lowest scaling equation estimates and our maximal models, although as with *Dreadnoughtus* there remains no overlap between the lowest scaling equation masses and those derived from the upper bounds of the mammalian convex hull expansion exponent (figure 2).

Convex hull volumes for extant taxa produced here required scaling exponents of between 1.18 and 1.91 (electronic supplementary material, tables S1–S6, and figures S8 and S9) to reach actual measured body masses, with three animals (American alligator 1.69; guineafowl 1.91; leghorn chicken 1.87) requiring exponents greater than that applied in our ‘maximal’ models (figure 1). However, increasing convex hull volume by 2.38, as required for our reconstruction of *Dreadnoughtus* to reach the mean scaling equation mass, results in substantial mass overestimates for all modelled extant taxa (23–102% overestimates; see electronic supplementary material, tables S1–S6).

Our analysis emphasizes a number of important points that should be considered in future studies. Firstly, it is vital that uncertainties and likely error magnitudes are explicitly acknowledged in mass estimates derived from all methods, including scaling equations. Our analysis also reveals that the higher range estimates predicted by bivariate scaling equations [3] appear to be highly incompatible with volumetric models that are based directly on currently available volume and density data from living vertebrates ([5]; electronic supplementary material, tables S1–S6). Indeed, in the case of *Dreadnoughtus*, the mean, and perhaps even some lower-end, scaling equation estimates appear to be implausible based on current data (figures 1 and 2). The high scaling equation mass for *Dreadnoughtus* also appears to result in a discrepancy in relative mass predictions between the modelled sauropods; our convex hull volumes (which provide a close approximation of the body volume defined by the preserved skeleton) of *Apatosaurus* and *Giraffatitan* represent 0.9 and 0.985 that of *Dreadnoughtus*, which appears congruent with the overlap in gross linear body proportions (electronic supplementary material, figure S11). By contrast, mean scaling equation mass predictions for *Apatosaurus* and *Giraffatitan* are 0.57 and 0.70 that of *Dreadnoughtus* (figure 2). While differences in skeletal : extra-skeletal dimensions should be expected [3], even in relatively closely related taxa (electronic supplementary material, tables S1–S6) it seems unlikely that differences in skeletal proportions of these three sauropods (figure 2; electronic supplementary material, figure S11) are sufficient to account for the 20–25 000 kg difference in body mass predicted by the scaling equation. Thus, even physiological and macroevolutionary studies that use relative mass values or distribute taxa into discrete mass ‘categories’ based on scaling equation estimates should take the maximum range of values or error inherent in these equations into account.

Recently, a similar pattern of divergence between volumetric and linear-based mass estimates was found for an exceptionally complete *Stegosaurus* skeleton [8]. The authors attributed this discrepancy to the ontogenetic status of the individual. Certain skeletal features may indicate that the *Dreadnoughtus* holotype was still growing at the time of death [2]. As an organism's body proportions change with age, the application of a scaling equation derived from modern adult skeletons to the limb bones of a sub- or young adult may be erroneous. At least some of the inconsistency we find here between mass estimation techniques may therefore be due to the ontogenetic stage of the specimen. Given the absence of confirmed ‘adult’ skeletal material for *Dreadnoughtus* however, it would be challenging to account for this phenomenon.

Estimating the mass of extinct animals is challenging [3,5,8–10]. By directly using the determinates of mass (volume and density) and maximizing skeletal evidence, volumetric approaches allow inherent uncertainties in mass predictions to be explicitly assessed (figures 1 and 2) and plausible limits established based on data and models of extant taxa. Our analysis reveals the importance of extending current analyses of dinosaur body mass in two ways; first and foremost by addition of further volumetric and density data on living taxa in order to more tightly constrain maximum plausible values for extinct animals. Second, a systematic comparison of dinosaur mass predictions from modelling and scaling equations, across a wide taxonomic and size range, is needed to identify and explain discrepancies between the two approaches (figure 2). Such a study would not only lead to more informed estimates of dinosaur body mass, but could also shed light on musculoskeletal adaptations for large body size in different dinosaur lineages.

## Supplementary Material

ElectronicSupplementaryMaterial

## Data accessibility

Convex hull models are downloadable from Dryad (http://dx.doi.org/10.5061/dryad.t5606).
