# Supplementary material for: Downsizing a giant: re-evaluating Dreadnoughtus body mass
Source: Biol Lett. 2015 Jun;11(6):20150215. doi: 10.1098/rsbl.2015.0215 (PMC4528471; doi:10.1098/rsbl.2015.0215)
Supplement: ElectronicSupplementaryMaterial [file rsbl20150215supp1.docx]

**Convex hull modeling and skeletal and volumetric reconstruction of *Dreadnoughtus***

The holotype of *Dreadnoughtus* is missing most of the cervical vertebra, as well the manus, skull and distal tip of the tail. There is also some taphonomic distortion to some skeletal elements, including severe crushing/deformation of the cervical vertebrae (see Lacovara et al [2] for discussion). Here we provide explicit explanation of how missing skeletal elements were accounted for in our model, and demonstrate how our minimum convex hulling approach minimizes both the level of subjective assumption required and also the magnitude of error resulting from skeletal incompleteness.

***Convex hulling and geometric completeness:*** Our approach to volumetric reconstruction involves tight-fitting 3D convex polygons to each body segment using a mathematical convex hulling algorithm [5,7]. As the extent of an object’s convex hull is dictated solely by its geometric extremes, the impact of missing skeletal elements is minimized: in other words, the resulting convex hull surrounding a skeletal body segment (e.g. neck segment, thoracic segment) will vary little - if at all - as a result of minor levels of incompleteness, as long as the overall body segment size and shape (as defined by its geometric extremes) are maintained. To demonstrate this we have generated a series of convex hull volumes around basic geometric shapes that have been subjected to increasing levels of random decimation (Figs S1-2). In the case of a sphere, the calculated convex hull volume remains above 98% the original value even when 80% of polygons making up that sphere have been removed (Figs S1-2). In cylindrical shapes the impact of decimation/incompleteness is even less significant: indeed, as long as the geometry of the circular ends of the cylinder are maintained there is absolutely no change in the volume of the convex hull volume (Figs S1-2). Pure vertical or lateral displacement of one end of the cylinder (i.e. as might occur to bone during taphonomic distortion) also results in no change to the convex hull volume (Figs S1-2). Decimation of the ends of the cylinder does result in some volume change (Figs S1-2).


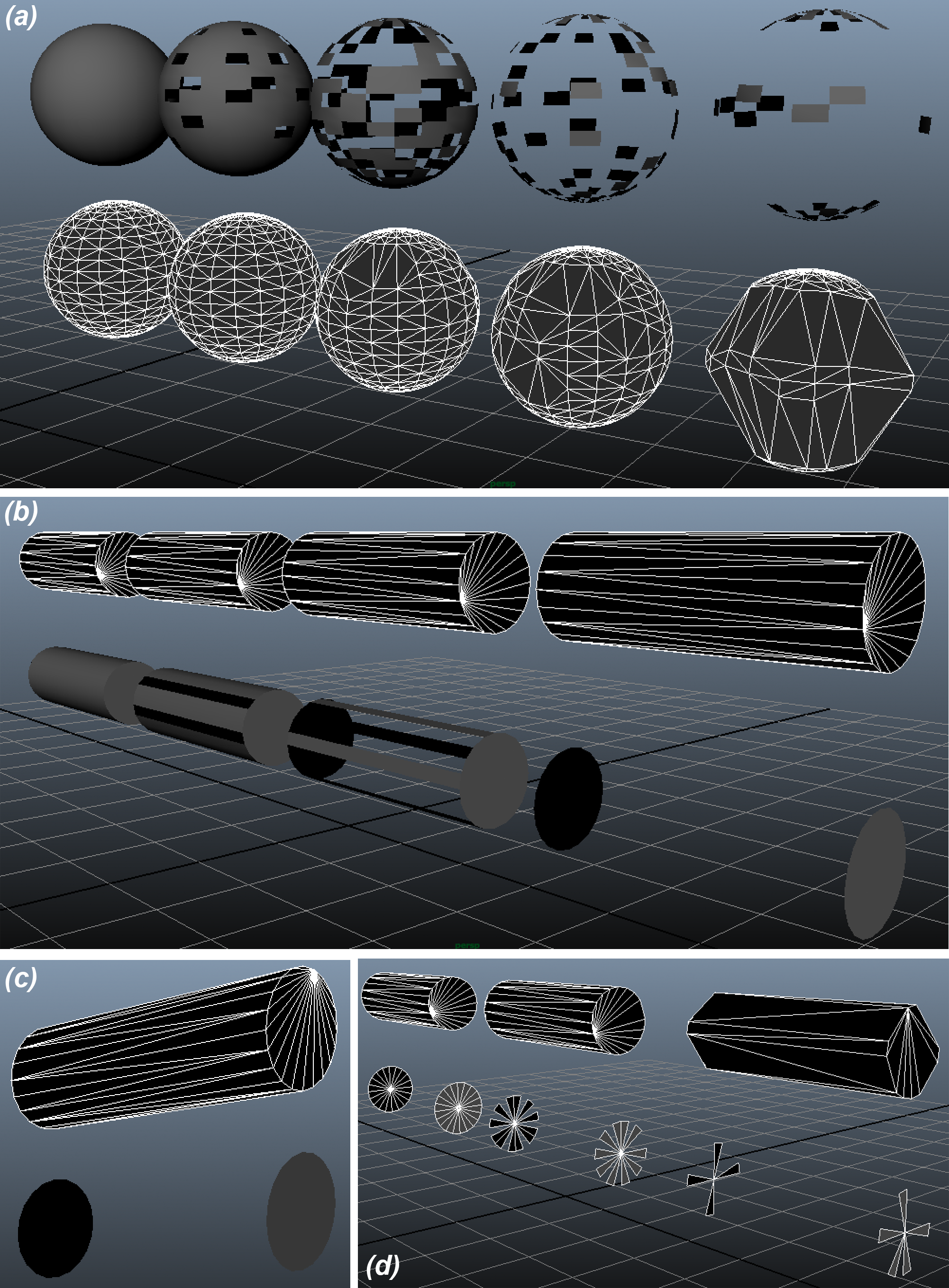


**Fig. S1.** Demonstration of the effect of object completeness on minimum convex hull volume reconstruction, using a series of increasingly randomly decimated (a) spheres and (b-d) cylinders. The quantitative impact of shape decimated is shown in Fig. S2.


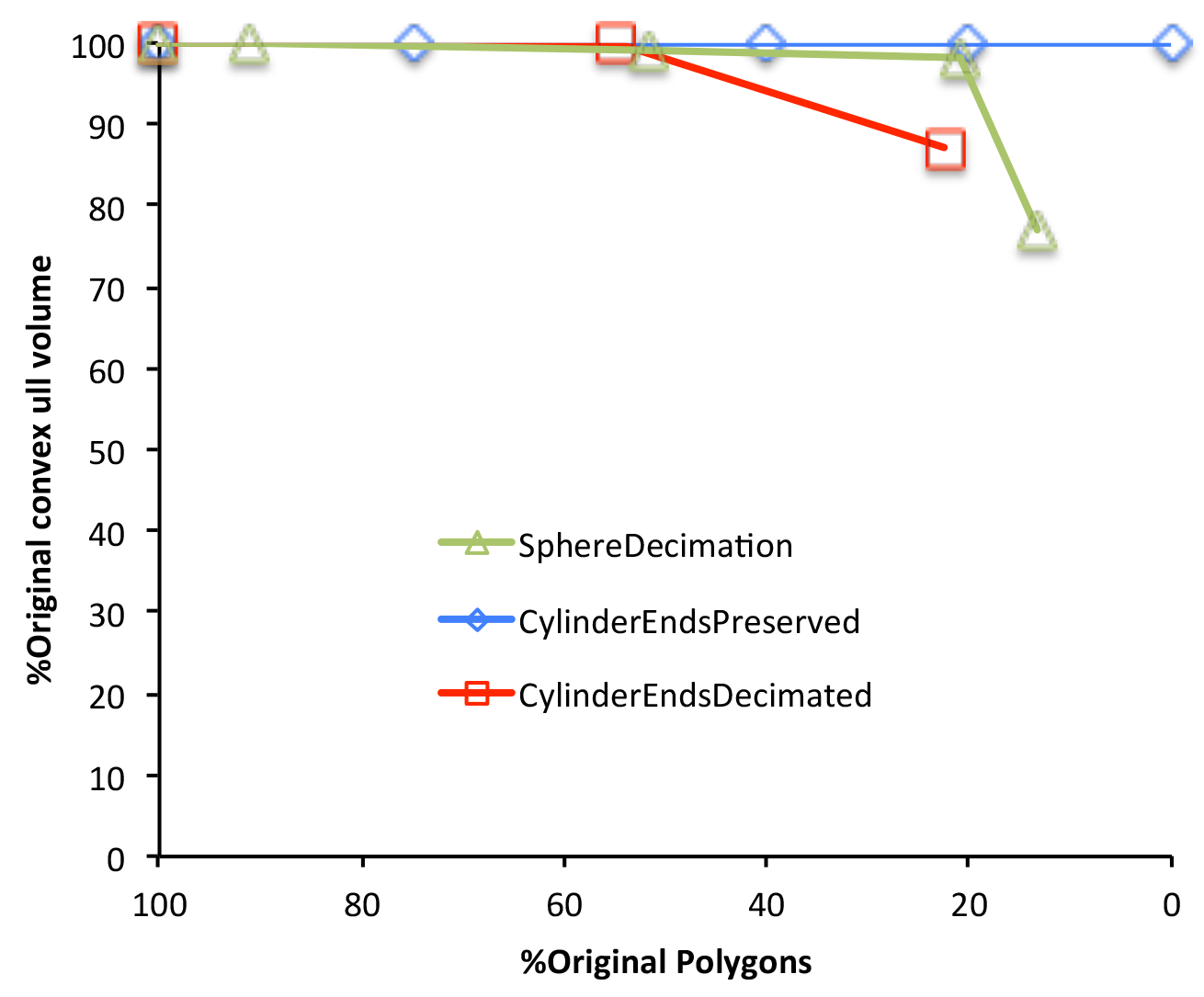


**Fig. S2.** Results from the shape decimation experiment shown in Fig. S1. The ‘SphereDecimation’ data series corresponds to the series of increasing decimated spheres shown in Fig. S1 (a), while ‘CylindersEndsPreserved’ corresponds to the cylinders depicted in Fig. S1 (b), and “CylindersEndsDecimated’ refers to cylinders shown in Fig. S1 (d).

To investigate the impact of skeletal completeness on minimum convex hull volumes generated for sauropod body segments we produced alternative reconstructions of the thoracic and neck segments in *Giraffatitan* (Figs S3-4). In the thoracic segment we deleted all but one mid-dorsal rib from the skeletal model, and in the neck segment we produced an alternative reconstruction using only the skeletal components preserved in *Dreadnoughtus* (see explanation of neck reconstruction below). Removing all but the single mid-dorsal rib made almost no difference to the resulting convex hull mass (19.75kg m3 versus 19.85 kg m3 in the original model; 0.49%) and by itself reduced the overall body volume by 0.004%. The ‘incomplete’ neck model, mimicking the reconstruction approach applied to *Dreadnoughtus*, resulting a larger relative change in neck volume; the incomplete neck model suffered a 9% decrease in volume relative to the original convex hull model. However, because of the much smaller size of the neck segment relative to the rest of the body segments, this represents only a 0.9% decrease in overall body volume. This leads us to conclude that skeletal incompleteness in *Dreadnoughtus* did not drastically affect the reconstructed convex hull volume, and subsequently our overall body mass predictions.


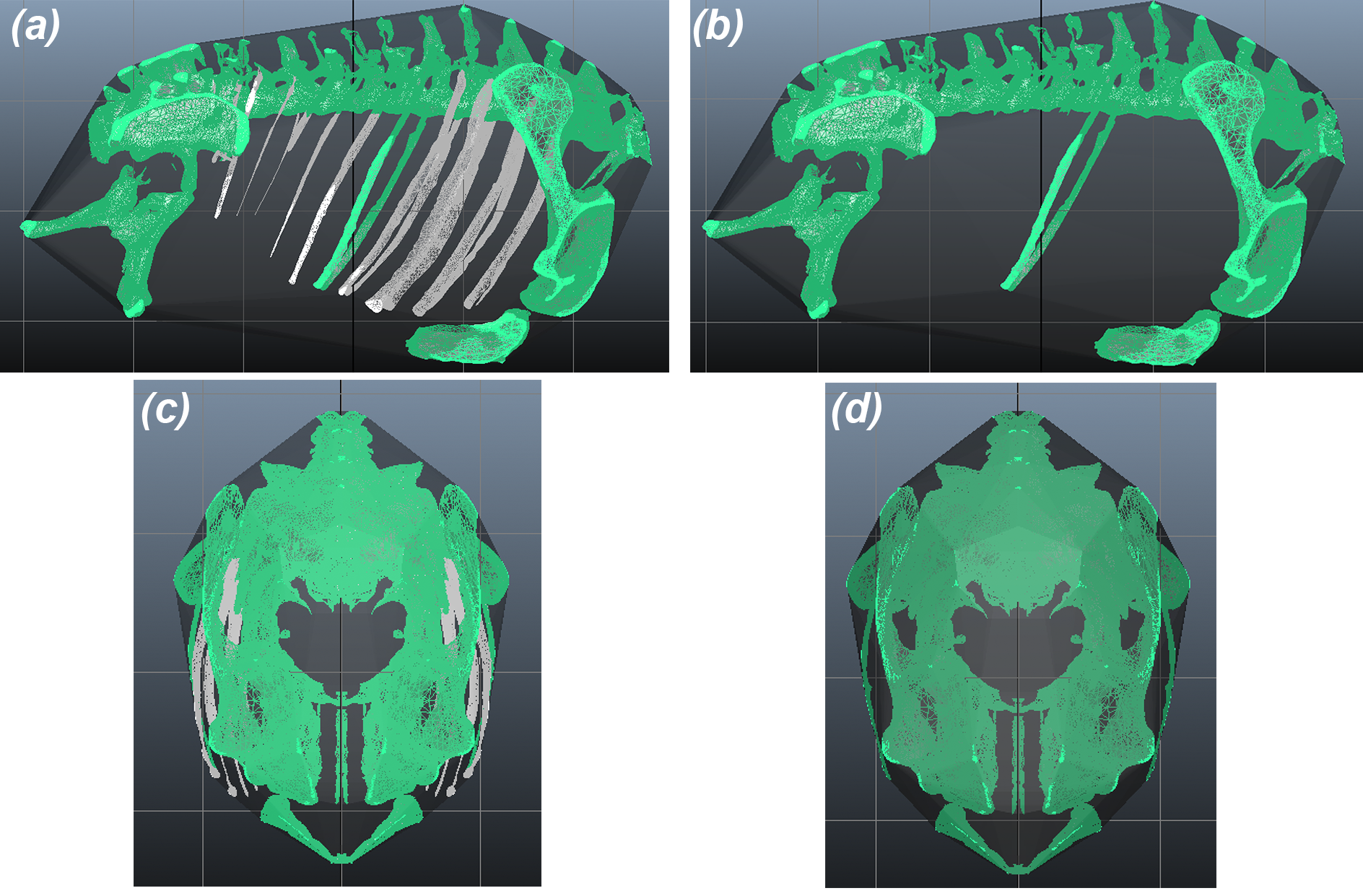


**Fig. S3.** Comparison of the minimum convex hull volumes generated around the ribcage, pectoral and pelvic girdles in *Giraffatitan* when the *(a & c)* full skeleton is used versus a model *(b & d )* in which only 1 full dorsal rib is retained (the deleted ribs are shown in white in *a, c & e*). *(a-b)* Show right lateral views and *(c-d)* cranial views.


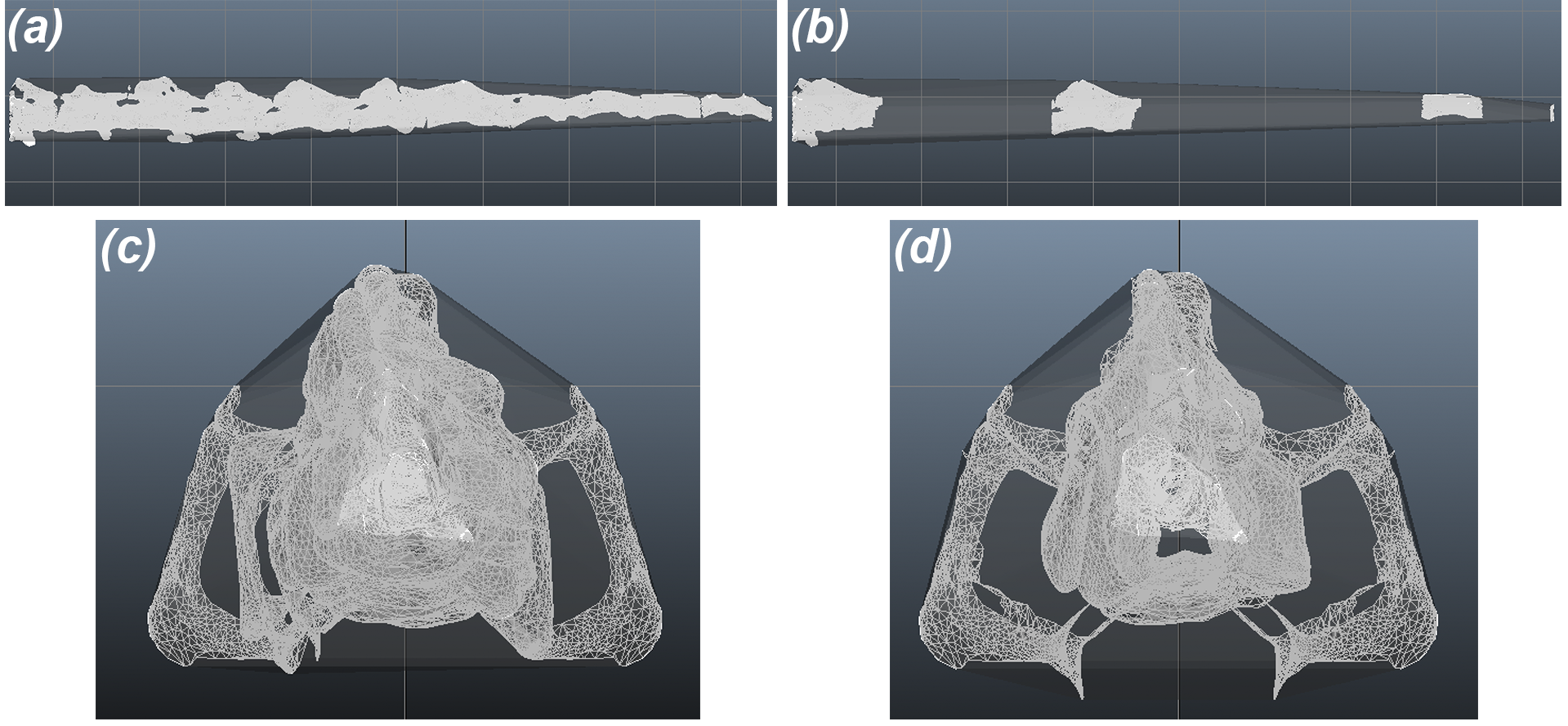


**Fig. S4.** Comparison of the minimum convex hull volumes generated for the neck segment in *Giraffatitan* when the *(a & c)* full skeleton is used versus a model *(b & d)* in which only the elements preserved in *Dreadnoughtus* are used*. (a-b)* Show right lateral views and *(c-d)* cranial views..

***Reconstructing*** ***Dreadnoughtus***: By reconstructing body volumes initially using minimum convex hulls we were able to minimize the amount of skeletal reconstruction in our model (Fig. S5). Prior to any skeletal reconstruction we generated minimum convex hulls around the preserved skeletal components in each body segment (Fig. S5 a-b & e-f). This yielded a total volume of 23.83m^3^, which equated to 88.5% of the volume of the restored full body convex hull reconstruction (Fig. S5 c-d & g-h), which was reconstructed as follows. For the manus and skull we used photogrammetric models of these elements from *Rapetosaurus*, another titanosaur, and re-scaled them using the reconstruction in Lacovara et al. [2, their Fig. 2]. To allow convex hulling to connect the ‘trunk’ and neck segments we duplicated the ninth cervical vertebra preserved in the specimen and placed its posterior surface above the most anterior point of pectoral girdle at a height consistent with the position of the preserved dorsal vertebrae. Finally we added an additional 10% on to the distal tail following the reconstruction of Lacovara et al. [2, their Fig. 2] as a guide.


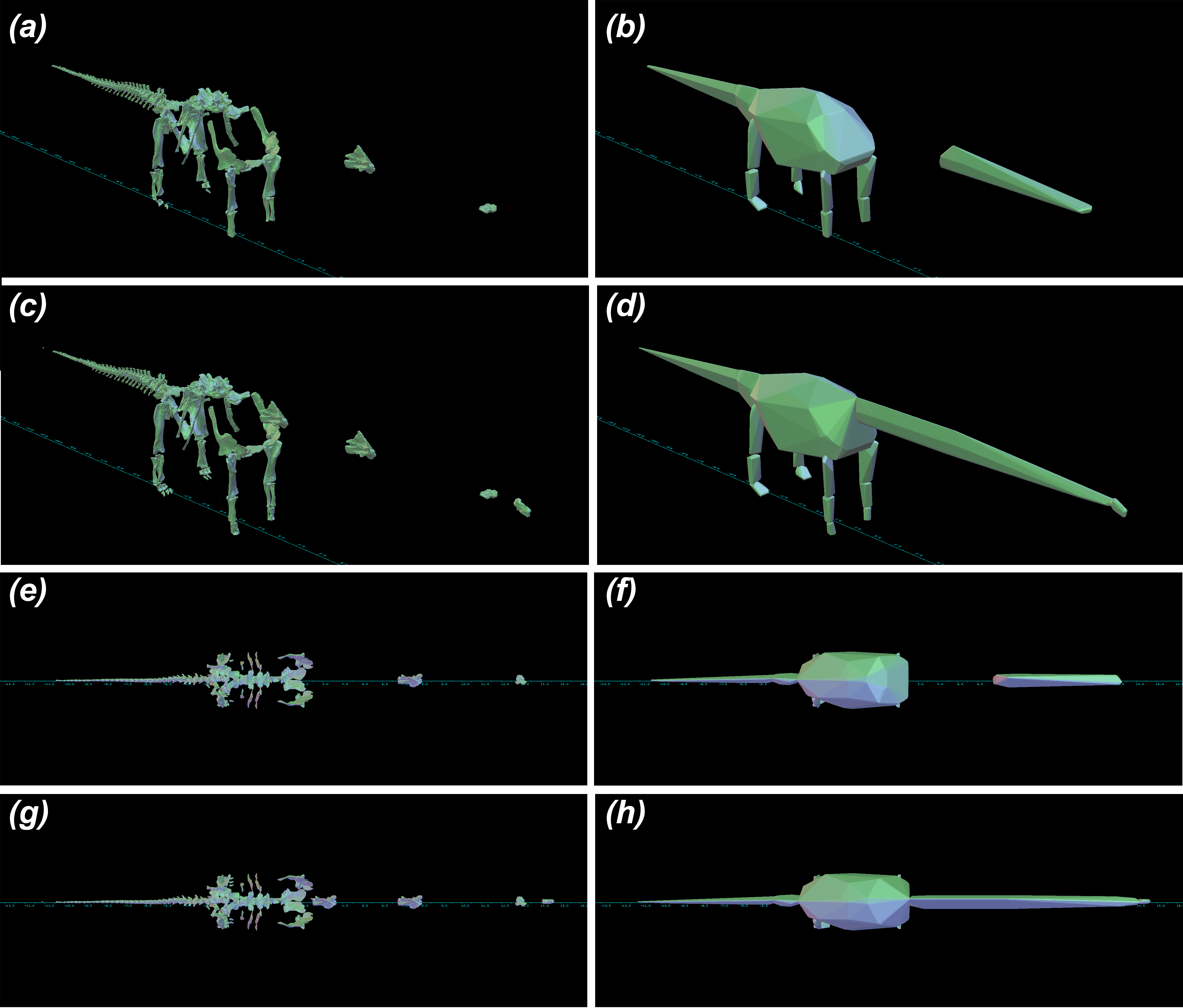


**Figure S5.** Skeletal and volumetric reconstruction of *Dreadnoughtus*, with explicit depiction of preserved and reconstructed skeletal components and their impact on the whole body convex hull reconstruction. ***(a & e)*** The preserved skeletal components and ***(b & f)*** convex hulls in generated around only those preserved components (in oblique and dorsal views), yielding a total volume of 23.83m^3^, which equates to 88.5% of the volume of full body convex hull reconstruction, shown in ***(d)*** and ***(h)***.

To assess the impact of taphonomic deformation on our volumetric reconstruction we altered the shape of the cervical vertebrae (the most deformed skeletal elements preserved). Specifically we deleted the highly deformed/crushed left sides of the preserved vertebrae and replaced them with a mirrored version of the right sides, which display much less severe taphonomic alteration (Fig. S6). The minimum convex volume generated around this modified neck reconstruction has a volume of 2.973kg m3, which is only 4.5% lower than that calculated without any correction for taphonomic deformation. This equates to a 0.5% reduction in overall body volume. We therefore retain use of the original bone scans of Lacovara et al. [2] without any retrodeformation, as any such process is likely introduce additional subjectivity without making any significant impact on the results.

**Additional model sensitivity experiments:** Two further experiments were carried out to determine the sensitivity of our predictions to poorly constrained aspects of sauropod dinosaur anatomy. Firstly, to further explore issues related to density and impact of zero-density respiratory structures on our *Dreadnoughtus* model we reconstructed a zero-density thoracic respiratory structure that filled the entre ribcage (Fig. S7). The volume of this structure is 9.129kg m3, which produces an overall increase of 212% over our original zero-density respiratory volume. This demonstrates that even with highly implausible reconstructions (Fig. S7) it is difficult to justify the extremely large zero-density respiratory volumes required to meet the ‘best-estimate’/mean scaling equation mass of 59,300kg while also maintaining whole-body densities similar to those estimated for sauropods.

Second, we tested for alternative reconstructions of neck length in *Dreadnoughtus* (Fig. S8). It is clear from the decimation analysis of simple shapes (Figs S1-2), our skeletal completeness analysis in *Giraffatitan* (Figs S3-4), and our retrodeformation analysis in *Dreadnoughtus* (Fig. S6) that modest size, and particularly shape differences do not result in large differences in the volumes of convex hulls. However, overall neck length in *Dreadnoughtus* is uncertain (see discussion above) and the initial reconstruction of Lacovara et al [2] used in our model is based on that of *Futalognkosaurus*. To test directly whether overall neck length has a major impact on overall volume/mass predictions we produced


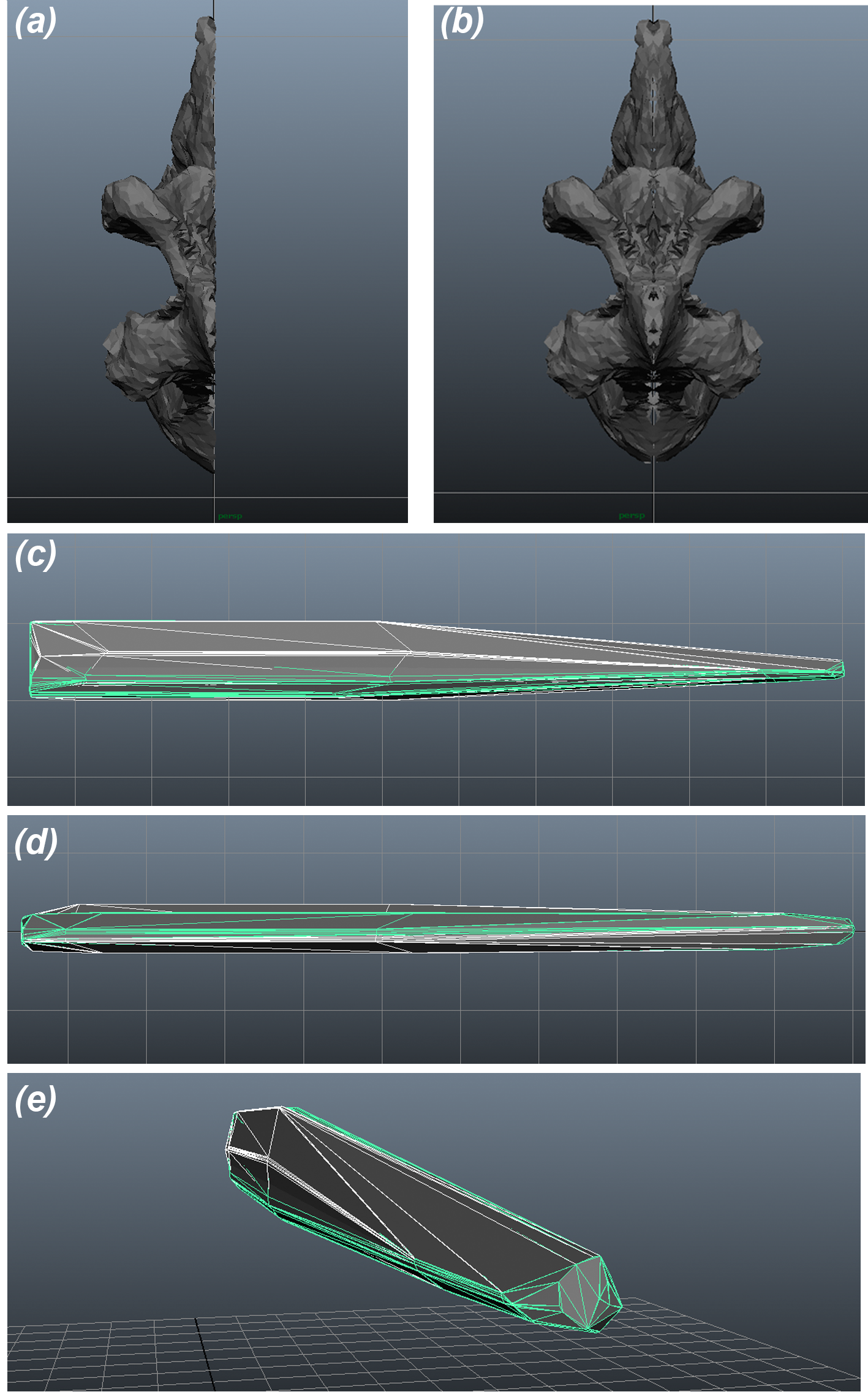


**Fig. S6.** The impact of taphonomic distortion and retrodeformation on the minimum convex hull volume of the neck segment in *Dreadnoughtus*. As a measure of the impact of taphonomic distortion on our reconstruction we ***(a)*** deleted the highly deformed/crushed left side of the cervical vertebrae (image shows 9^th^ cervical in cranial view) and ***(b)*** digitally mirrored the remaining right side, and finally ***(c-e)*** regenerated the minimum convex hull volume for the neck. ***(c-e)*** The original ‘uncorrected’ convex hull volume in white, and ‘retrodeformed’ volume in green in ***(c)*** right lateral, ***(d)*** dorsal and ***(e)*** cranio-lateral views.


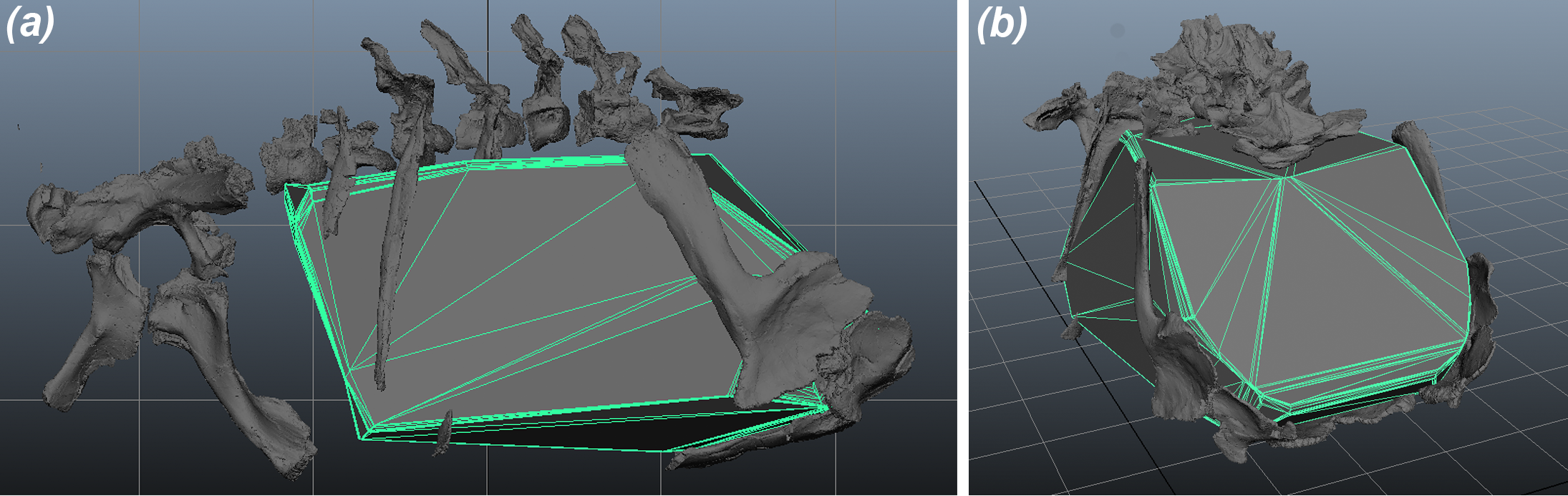


**Fig. S7.** Zero-density thoracic respiratory structure filling the entire ribcage of *Dreadnoughtus*.

three additional neck reconstructions, in which overall neck length was decreased by 10%, and increased by 10% and 20% (Fig. S8). These decreases/increases were achieved by increasing the spacing between each preserved vertebra by equal amounts, which will have a larger (i.e. worst case scenario) impact on convex volume than increasing neck length by simply displacing only the most cranial vertebrae. Decreasing and increasing neck length by 10% yielded a 9% change in neck volume relative to the original convex hull model, with 20% increase in neck length producing an 18% increase in neck volume. However, the impact on overall whole-body volume is extremely modest: the +/-10% neck length models alter whole-body volume by approximately 1%, with the 20% increase model yielding an approximately 2% increase in whole-body volume. We are therefore confident that overall neck length (Fig. S8), and neck geometry (Figs S4&6) do not have an important impact on our discussion and comparison of model and scaling equation masses in the main text.

**
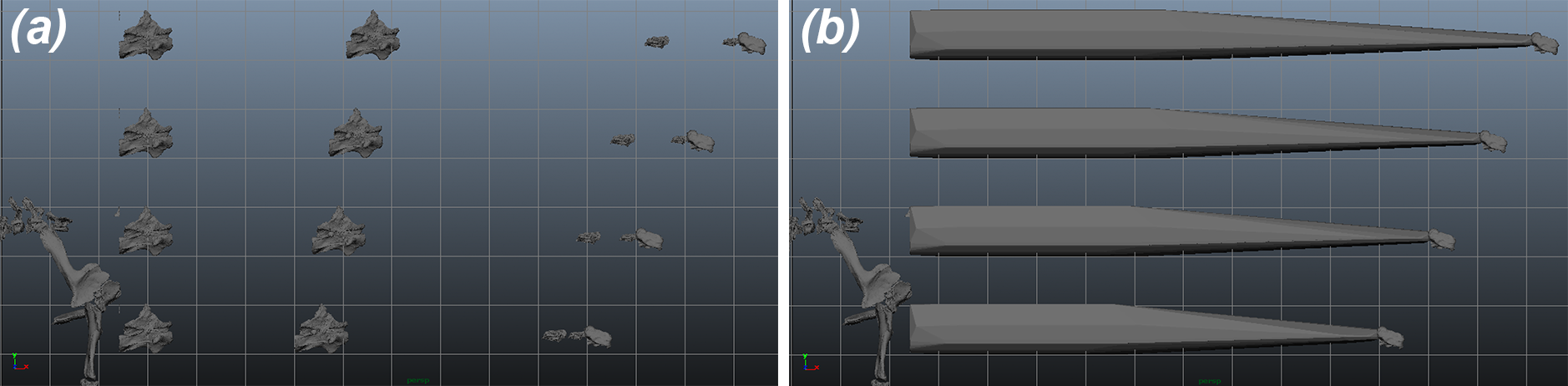
**

**Fig. S8.** Alternative neck length (a) skeletal and (b) convex hull volume reconstructions in *Dreadnoughtus*. From top to bottom reconstructions are: Plus 20%, Plus 10%, original reconstruction (based on Lacovara et al [2]) and Minus 10% overall neck length.

***Volumetric analysis of extant animals:*** The volumetric data generated for the six extant animals (American alligator, Australian saltwater crocodile, a varanid lizard, two different breeds of chickens and a guineafowl) are tabulated below in Tables S1-6. In each case the specimens were digitized with medical grade CT scanners and 3D skeletal models generated by image segmentation in either Mimics (in the case of *Crocodylus* and the junglefowl, which are from [10]) or Avizo. The Australian saltwater crocodile and junglefowl data comes from a previously published study of archosaur mass properties [10], while the *Varanus* data comes from Schachner et al [11]. The American alligator and leghorn chicken were both sub-adults and were farmed specimens. The images below show example volumes for the (Fig. S9) American alligator and (Fig. S10) leghorn chicken models.


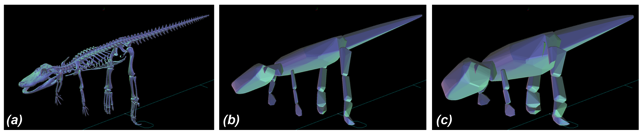


**Figure S9.** Examplar images of the Alligator (*Alligator* *mississippiensis*) ***(a)*** 3D skeletal model, ***(b)*** convex hull volumes and ***(c)*** convex hull volumes expanded by 2.38 times.


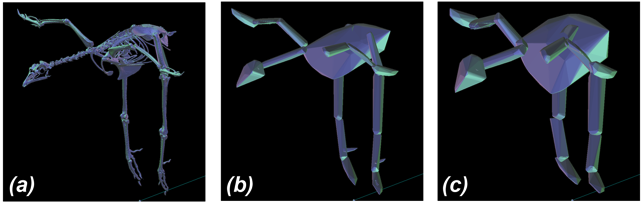


**Figure S10**. Leghorn chicken (*Gallus gallus*) ***(a)*** 3D skeletal model, ***(b)*** convex hull volumes and ***(c)*** convex hull volumes expanded by 2.38 times.

***Sauropod body proportions:*** Fig. S11 shows a number of measures of gross body proportions in *Giraffatitan*, *Apatosaurus* and *Dreadnoughtus*, emphasizing that whilst they have different body shapes (e.g. longer neck in *Dreadnoughtus*, longer tail in *Apatosaurus*) they share broadly similar overall skeletal proportions, which appears at odds with the 20-25,000kg difference in body mass predicted by scaling equations (Fig. 2).


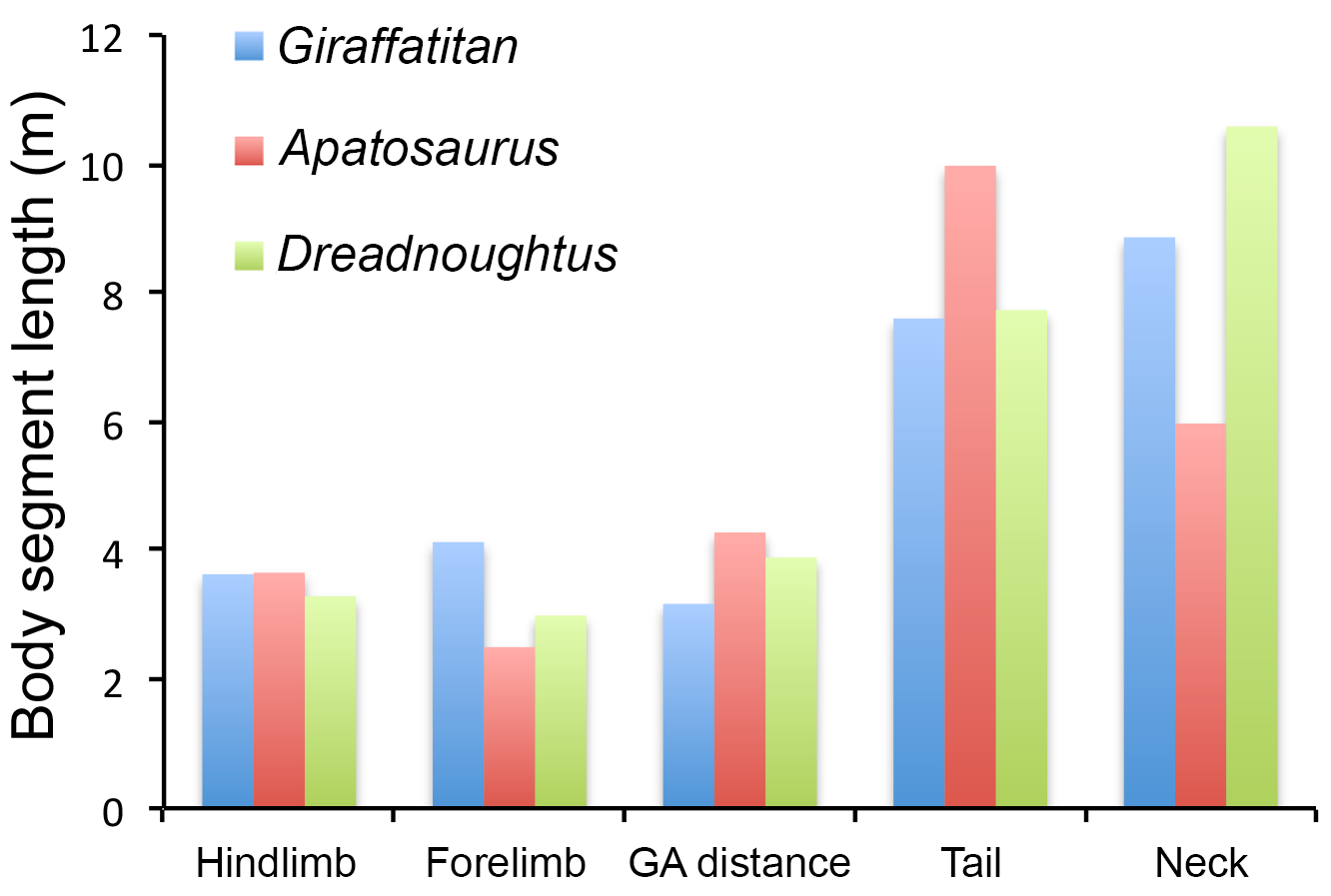


Fig. S11. Gross body proportions measured from the 3D models of *Giraffatitan*, *Apatosaurus* and *Dreadnoughtus*. GA, glenoacetabular.

**Table S1.** Convex hull body segment volumes and masses for *Alligator mississippiensis* specimen. The density used is that of an adult *Crocodylus* *johnstoni* [10]. Volumes in m^3^, Densities in kg m^3^ and masses in kg.

|  | Actual Mass (kg) | 10.8 |  |  |
| --- | --- | --- | --- | --- |
|  |  | | |  |
|  | Volume | Density | Mass |  |
| Head | 0.001192 | 948 | 1.130 |  |
| Neck | 0.000128 | 948 | 0.121 |  |
| Trunk | 0.003996 | 948 | 3.788 |  |
| Tail | 0.000647 | 948 | 0.613 |  |
| Humerus | 0.000024 | 948 | 0.023 |  |
| Forearm | 0.000014 | 948 | 0.013 |  |
| Hand | 0.000016 | 948 | 0.015 |  |
| Humerus | 0.000024 | 948 | 0.023 |  |
| Forearm | 0.000014 | 948 | 0.013 |  |
| Hand | 0.000016 | 948 | 0.015 |  |
| Thigh | 0.000108 | 948 | 0.102 |  |
| Shank | 0.000111 | 948 | 0.105 |  |
| Metatarsals | 0.000051 | 948 | 0.048 |  |
| Foot | 0.000049 | 948 | 0.046 |  |
| Thigh | 0.000108 | 948 | 0.102 |  |
| Shank | 0.000111 | 948 | 0.105 |  |
| Metatarsals | 0.000051 | 948 | 0.048 |  |
| Foot | 0.000049 | 948 | 0.046 |  |
| **Axial total** | **0.005963** | **948** | **5.653** |  |
| **Hind limb total** | **0.000319** | **948** | **0.302** |  |
| **Fore limb total** | **0.000054** | **948** | **0.051** |  |
|  |  |  |  |  |
| **Model Iterations** | **Volume** | **Density** | **Mass** | **%Actual mass** |
| Convex Hull | 0.006709 | 948 | 6.360 | 59 |
| Plus21% | 0.00811789 | 948 | 7.6958 | 71 |
| Maximal | 0.01080149 | 948 | 10.2398 | 95 |
| *Dreadnoughtus* Exponent | 0.01596742 | 948 | 15.1371 | 140 |

**Table S2.** Convex hull body segment volumes and masses for *Crocodylus* *johnstoni*. The density used is that of an adult *Crocodylus* *johnstoni* [10]. Volumes in m^3^, Densities in kg m^3^ and masses in kg.

|  | Actual Mass(kg) | 20.19 |  |  |
| --- | --- | --- | --- | --- |
|  |  |  |  |  |
|  | Volume | Density | Mass |  |
| Head | 0.00172134 | 948 | 1.632 |  |
| Neck | 0.000406 | 948 | 0.385 |  |
| Trunk | 0.008491 | 948 | 8.049 |  |
| Tail | 0.001932 | 948 | 1.832 |  |
| Humerus | 0.00007 | 948 | 0.066 |  |
| Forearm | 0.000043 | 948 | 0.041 |  |
| Hand | 0.000032 | 948 | 0.030 |  |
| Humerus | 0.00007 | 948 | 0.066 |  |
| Forearm | 0.000043 | 948 | 0.041 |  |
| Hand | 0.000032 | 948 | 0.030 |  |
| Thigh | 0.000076 | 948 | 0.072 |  |
| Shank | 0.000108 | 948 | 0.102 |  |
| Foot | 0.000058 | 948 | 0.055 |  |
| Thigh | 0.000076 | 948 | 0.072 |  |
| Shank | 0.000108 | 948 | 0.102 |  |
| Foot | 0.000058 | 948 | 0.055 |  |
| **Axial Total** | **0.012550** | 948 | 11.898 |  |
| **Hind limb Total** | **0.000484** | 948 | 0.459 |  |
| **Fore limb Total** | **0.000290** | 948 | 0.275 |  |
|  |  |  |  |  |
| **Model Iterations** | **Volume** | **Density** | **Mass** | **%Actual mass** |
| Convex Hull | 0.013324 | 948 | 12.631 | 63 |
| Plus21% | 0.016122 | 948 | 15.284 | 76 |
| Maximal | 0.021452 | 948 | 20.337 | 101 |
| *Dreadnoughtus* Exponent | 0.031712 | 948 | 30.063 | 149 |

**Table S3.** Convex hull body segment volumes and masses for *Varanus* *exanthematicus*. The density used is that of an adult *Crocodylus* *johnstoni* [10]. Volumes in m^3^, Densities in kg m^3^ and masses in kg.

|  | Actual Mass (kg) | 0.597 |  |  |
| --- | --- | --- | --- | --- |
|  |  |  |  |  |
|  | Volume | Density | Mass |  |
| Head | 0.0000372867 | 948 | 0.035 |  |
| Neck | 0.0000057135 | 948 | 0.005 |  |
| Trunk | 0.0004358131 | 948 | 0.413 |  |
| Tail | 0.0000188503 | 948 | 0.018 |  |
| Humerus | 0.0000027937 | 948 | 0.003 |  |
| Forearm | 0.0000017546 | 948 | 0.002 |  |
| Hand | 0.0000044318 | 948 | 0.004 |  |
| Humerus | 0.0000027937 | 948 | 0.003 |  |
| Forearm | 0.0000017546 | 948 | 0.002 |  |
| Hand | 0.0000044318 | 948 | 0.004 |  |
| Thigh | 0.0000021662 | 948 | 0.002 |  |
| Shank | 0.0000028120 | 948 | 0.003 |  |
| Foot | 0.0000049809 | 948 | 0.005 |  |
| Thigh | 0.0000021662 | 948 | 0.002 |  |
| Shank | 0.0000028120 | 948 | 0.003 |  |
| Foot | 0.0000049809 | 948 | 0.005 |  |
| **Axial Total** | **0.0004976637** | 948 | 0.472 |  |
| **Hind limb Total** | **0.0000199183** | 948 | 0.019 |  |
| **Fore limb Total** | **0.0000179602** | 948 | 0.017 |  |
|  |  |  |  |  |
| **Model Iterations** | **Volume** | **Density** | **Mass** | **%Actual mass** |
| Convex Hull | 0.000536 | 948 | 0.508 | 85 |
| Plus21% | 0.000648 | 948 | 0.614 | 103 |
| Maximal | 0.000862 | 948 | 0.817 | 137 |
| *Dreadnoughtus* Exponent | 0.001275 | 948 | 1.208 | 202 |

**Table S4.** Convex hull body segment volumes and masses for *Gallus gallus (leghorn)*. The density used is that of an adult junglefowl chicken [10]. Volumes in m^3^, Densities in kg m^3^ and masses in kg.

|  | Actual Mass (kg) | 1.15 |  |  |
| --- | --- | --- | --- | --- |
|  |  |  |  |  |
|  | Volume | Density | Mass |  |
| Head | 0.00002652 | 894 | 0.023706556 |  |
| Neck | 0.00001497 | 894 | 0.013384074 |  |
| Trunk | 0.00049388 | 894 | 0.441525144 |  |
| Humerus | 0.00001087 | 894 | 0.009716439 |  |
| Forearm | 0.00000791 | 894 | 0.007071352 |  |
| Hand | 0.00000450 | 894 | 0.004018834 |  |
| Humerus | 0.00001087 | 894 | 0.009716439 |  |
| Forearm | 0.00000791 | 894 | 0.007071352 |  |
| Hand | 0.00000450 | 894 | 0.004018834 |  |
| Thigh | 0.00001108 | 894 | 0.009906682 |  |
| Shank | 0.00002388 | 894 | 0.021344429 |  |
| Metatarsals | 0.00001128 | 894 | 0.010082582 |  |
| Foot | 0.00000722 | 894 | 0.006457273 |  |
| Thigh | 0.00001108 | 894 | 0.009906682 |  |
| Shank | 0.00002388 | 894 | 0.021344429 |  |
| Metatarsals | 0.00001128 | 894 | 0.010082582 |  |
| Foot | 0.00000722 | 894 | 0.006457273 |  |
| **Axial total** | **0.00053536** | **894** | **0.479** |  |
| **Hind limb total** | **0.00005346** | **894** | **0.048** |  |
| **Fore limb total** | **0.00002327** | **894** | **0.021** |  |
|  |  |  |  |  |
| **Model Iterations** | **Volume** | **Density** | **Mass** | **%Actual mass** |
| Convex Hull | 0.0006888 | 894 | 0.616 | 54 |
| Plus21% | 0.00083348 | 894 | 0.7451 | 65 |
| Maximal | 0.00110901 | 894 | 0.9915 | 86 |
| *Dreadnoughtus* Exponent | 0.00163941 | 894 | 1.4656 | 127 |

**Table S5.** Convex hull body segment volumes and masses for the Guineafowl. The density used is that of an adult junglefowl chicken [10]. Volumes in m^3^, Densities in kg m^3^ and masses in kg.

|  | Actual Mass (kg) | 1.352 |  |  |
| --- | --- | --- | --- | --- |
|  |  |  |  |  |
|  | Volume | Density | Mass |  |
| Head | 0.00002602 | 894 | 0.0233 |  |
| Neck | 0.00001781 | 894 | 0.0159 |  |
| Trunk | 0.00059135 | 894 | 0.5287 |  |
| Humerus | 0.00001277 | 894 | 0.0114 |  |
| Forearm | 0.00000850 | 894 | 0.0076 |  |
| Hand | 0.00000374 | 894 | 0.0033 |  |
| Humerus | 0.00001277 | 894 | 0.0114 |  |
| Forearm | 0.00000850 | 894 | 0.0076 |  |
| Hand | 0.00000374 | 894 | 0.0033 |  |
| Thigh | 0.00001561 | 894 | 0.0140 |  |
| Shank | 0.00002128 | 894 | 0.0190 |  |
| Metatarsals | 0.00000630 | 894 | 0.0056 |  |
| Foot | 0.00000657 | 894 | 0.0059 |  |
| Thigh | 0.00001561 | 894 | 0.0140 |  |
| Shank | 0.00002128 | 894 | 0.0190 |  |
| Metatarsals | 0.00000630 | 894 | 0.0056 |  |
| Foot | 0.00000657 | 894 | 0.0059 |  |
| **Axial Total** | **0.00063517** | **894** | **0.5678** |  |
| **Hind limb Total** | **0.00009953** | **894** | **0.0890** |  |
| **Fore limb Total** | **0.00005003** | **894** | **0.0447** |  |
|  |  |  |  |  |
| **Model Iterations** | **Volume** | **Density** | **Mass** | **%Actual mass** |
| Convex Hull | 0.00078473 | 894 | 0.7015 | 52 |
| Plus21% | 0.00094952 | 894 | 0.8489 | 63 |
| Maximal | 0.00126341 | 894 | 1.1295 | 84 |
| *Dreadnoughtus* Exponent | 0.00186765 | 894 | 1.6697 | 123 |

**Table S6.** Convex hull body segment volumes and masses for *Gallus gallus (junglefowl)*. The density used is that of an adult *junglefowl* [10]. Volumes in m^3^, Densities in kg m^3^ and masses in kg.

|  | Actual Mass (kg) | 1.94 |  |  |
| --- | --- | --- | --- | --- |
|  |  |  |  |  |
|  | Volume | Density | Mass |  |
| Head | 0.000041 | 894 | 0.036654 |  |
| Neck | 0.000033 | 894 | 0.029502 |  |
| Trunk | 0.001001 | 894 | 0.894894 |  |
| Humerus | 0.000032 | 894 | 0.028608 |  |
| Forearm | 0.000034 | 894 | 0.030396 |  |
| Hand | 0.000114 | 894 | 0.101916 |  |
| Humerus | 0.000032 | 894 | 0.028608 |  |
| Forearm | 0.000034 | 894 | 0.030396 |  |
| Hand | 0.000114 | 894 | 0.101916 |  |
| Thigh | 0.000046 | 894 | 0.041124 |  |
| Shank | 0.000065 | 894 | 0.05811 |  |
| Metatarsals | 0.000021 | 894 | 0.018774 |  |
| Foot | 0.000032 | 894 | 0.028608 |  |
| Thigh | 0.000046 | 894 | 0.041124 |  |
| Shank | 0.000065 | 894 | 0.05811 |  |
| Metatarsals | 0.000021 | 894 | 0.018774 |  |
| Foot | 0.000032 | 894 | 0.028608 |  |
| **Axial Total** | **0.00107500** | 894 | 0.96105 |  |
| **Hind limb Total** | **0.00032800** | 894 | 0.293232 |  |
| **Fore limb Total** | **0.00036000** | 894 | 0.32184 |  |
|  |  |  |  |  |
| **Model Iterations** | **Volume** | **Density** | **Mass** | **%Actual mass** |
| Convex Hull | 0.00176300 | 894 | 1.5761 | 81 |
| Plus21% | 0.00213323 | 894 | 1.9071 | 98 |
| Maximal | 0.00283843 | 894 | 2.5376 | 131 |
| *Dreadnoughtus* Exponent | 0.00419594 | 894 | 3.7512 | 193 |

Table S7. Previously published density estimates for sauropod dinosaurs.

| **Source** | **Whole body density estimate (kg m^-3^)** | **Segment densities modelled** |
| --- | --- | --- |
| [12] | 900 | NA - homogenous density applied |
| [13] | 900 | Lungs estimated at 10% body volume, subtracted from whole estimate |
| [14] | 900 | NA - homogenous density applied (based on [13]) |
| [15] | Not stated | Neck: 600 kgm^-3^ ; 'Main body': 900 kgm^-3^ |
| [16] | 900 | Estimated lung volume subtracted from whole body volume, both at 1000kgm^-3^ |
| [8] | 791-818  (mean densities for 4 taxa) | Axial: 1000 kgm^-3^ ; Trunk: 850kgm^-3^ (accounting for lungs & air sacs) ; Neck: 600 kgm^-3^ ; Limbs: 1050 kgm^-3^ |
| [17] | 800 | Extraskeletal diverticulae, trachea, lungs & air sacs modelled; their volumes subtracted  from whole body volume |
| [18] | Not stated | Trunk: 800 kgm^-3^ ; Neck: 300 kgm^-3^ ; Limbs & Tail: 1000 kgm^-3^ |
| [19] | 800 | NA - homogenous density applied (based on [17]) |
| [20] | 800 | NA - homogenous density applied (based on [17]) |

**Full Reference List**

1. Benson, R. B. J., Campione, N. E., Carrano, M. T., Mannion, P. D., Sullivan, C., Upchurch, P. & Evans, D. C. 2014. Rates of Dinosaur Body Mass Evolution Indicate 170 Million Years of Sustained Ecological Innovation on the Avian Stem Lineage. *PLoS Biology*, doi:10.1371/journal.pbio.1001853.
2. Lacovara, K. J. et al. 2014. A Gigantic, Exceptionally Complete Titanosaurian Sauropod Dinosaur from Southern Patagonia, Argentina. *Scientific Reports* **4**, doi:10.1038/srep06196.
3. Campione, N. E. & Evans, D. C. 2012. A universal scaling relationship between body mass and proximal limb bone dimensions in quadrupedal terrestrial tetrapods. *BMC Biology* **10**,  doi:10.1186/1741-7007-10-60.
4. Taborda, J. R. A., Cerda, I. A. & Desoio, J. B. 2013. Growth curve of *Aetosauroides scagliai* Casamiquela 1960 (Pseudosuchia: Aetosauria) inferred from osteoderm histology. *Geol. Soc. Lond. Special Publications* **379**, 413-423.
5. Sellers. W. I., Hepworth-Bell, J., Falkingham, P. L., Bates, K. T., Brassey, C., Egerton, V. & Manning, P. L. 2012. Minimum convex hull mass estimations of complete mounted skeletons. *Biology Letters* **8**, 842-845.
6. Falkingham, P. L. 2012. Acquisition of high resolution 3D models using free, open-source, photogrammetric software. *Palaeontologia Electronica* **15**, 1; 1T:15p
7. Brassey, C., Maidment, S. C. & Barrett, P. M. 2014. Body mass estimates of an exceptionally complete Stegosaurus (Ornithischia: Thyreophora): comparing volumetric and linear bivariate mass estimations methods. Biology Letters.
8. Henderson DM (2004) Tipsy punters: Sauropod dinosaur pneumaticity, buoyancy and aquatic habits. *Proc. Roy. Soc. B,* **271,** S180-S183.
9. Bates, K. T., Manning, P. L., Hodgetts, D. and Sellers, W. I. 2009a. Estimating mass properties of dinosaurs using laser imaging and computer modeling. *PLoS ONE,* **4**, doi:10.1371/journal.pone.0004532.
10. Allen, V., Paxton, H. & Hutchinson, J.R. 2009. Variation in center of mass estimates for extant sauropsids and its importance for reconstructing inertial properties of extinct archosaurs. The Anatomical Record **292**, 9, 1442-1461.
11. Schachner, E. R., Cieri, R. L., Butler, J. P. & Farmer, C. G. 2013. Unidirectional pulmonary airflow patterns in the savannah monitor lizard. Nature doi:10.1038/nature12871.
12. Colbert EH (1962) The Weights of Dinosaurs. *American Museum Noviates,* **2076,** 1-16.
13. Alexander RM (1985) Mechanics of posture and gait of some large dinosaurs. *Zoological Journal of the Linnean Society,* **83,** 1-25.
14. Gunga HC, Kirsch KA, Baartz F, et al. (1995) New data on the dimensions of Brachiosaurus brancai and their physiological implications. *Naturwissenschaften,* **82,** 190-192.
15. Paul GS (1997) Dinosaur Models: The Good, The Bad, and Using Them to Estimate the Mass of Dinosaurs. *Dinofest International Proceedings***,** 129-142.
16. Henderson DM (1999) Estimating the masses and centers of mass of extinct animals by 3-D mathematical slicing. Paleobiology, **25**, 88-106.
17. Henderson DM (2006) Burly gaits; centers of mass, stability, and the trackways of sauropod dinosaurs. Journal of Vertebrate Paleontology, **26**, 907-921.
18. Wedel M. J. 2005 Postcranial skeletal pneumaticity in sauropods and its implications for mass estimates. In *The sauropods: evolution and paleobiology* (eds Wilson J. A., Curry-Rogers K.), pp. 201–228. Berkeley, CA: University of California Press.
19. Taylor MP (2009) A re-evaluation of Brachiosaurus altithorax Riggs 1903 (Dinosauria, Sauropoda) and its generic separation from *Giraffatitan brancai* (Janensch 1914). Journal of Vertebrate Paleontology, **29**, 787-806.
20. Gunga H-C, Suthau T, Bellmann A, et al. (2008) A new body mass estimation of Brachiosaurus brancai Janensch, 1914 mounted and exhibited at the Museum of Natural History (Berlin, Germany). Fossil Record, **11**, 33-38.
